# Isolation, Identification, and Prophylactic Evaluation of a Newly Isolated *C. perfringens* Phage

**DOI:** 10.3390/microorganisms14081683

**Published:** 2026-07-31

**Authors:** Hui Wang, Qihuan Zhao, Bo Wang, Chengquan Du, Jingjing Wang, Liang Zhang, Fuxiang Bao

**Affiliations:** 1 College of Veterinary Medicine, Inner Mongolia Agricultural University, Hohhot 010010, China; wanghui2024@emails.imau.edu.cn (H.W.); zhaoqihuan@emails.imau.edu.cn (Q.Z.); wangboemail@emails.imau.edu.cn (B.W.); ducq@emails.imau.edu.cn (C.D.); ggble@emails.imau.edu.cn (J.W.); 2 Key Laboratory of Clinical Diagnosis and Treatment Technology for Animal Diseases, Ministry of Agriculture and Rural Affairs, Hohhot 010010, China

**Keywords:** *C. perfringens*, phage, phage therapy, mouse model

## Abstract

*Clostridium perfringens* is a major livestock pathogen responsible for histotoxic diseases and intestinal infections, posing serious threats to public health and the poultry and breeding industry. Overreliance on antibiotics has driven the emergence of drug-resistant strains and disruption of gut microbiota homeostasis, underscoring the urgent need for alternative control strategies. In this study, a lytic phage designated vB_CppII_Hohhot was isolated from wastewater samples collected from large-scale dairy farms in Inner Mongolia. The optimal multiplicity of infection was determined to be 0.1. pH stability was assessed. The phage was found to remain stable across pH 3 to 11, but no viable phage was detected at pH 12 (detection limit: <1 PFU/mL). Temperature adaptability was also assessed. Peak titers were achieved between 37°C and 40°C, and the titer was observed to fall below the detection limit at temperatures above 45°C. At a constant temperature of 37 °C, the phage rapidly reached the plateau phase within 40 min, with bacterial counts significantly reduced by 14 h. Whole-genome sequencing revealed that the phage genome consisted of 76 open reading frames, with no detectable antibiotic resistance or virulence genes; an endolysin gene potentially responsible for its lytic activity was also identified. In a prophylactic mouse model, phage administration significantly reduced pathological damage. Collectively, these findings provide a newly isolated candidate agent and a theoretical basis for the prophylactic application of phages against *C. perfringens*-associated infections.

## 1. Introduction

*Clostridium perfringens* (*C. perfringens*) is a Gram-positive, anaerobic, spore-forming bacterium that is widely distributed in nature [1], and transmitted through contaminated feed or soil. Infections can also be induced by imbalances in the intestinal microbiota, causing severe intestinal disorders such as necrotic enteritis and enterotoxemia, particularly threatening calves and other domestic animals [2]. This bacterium impairs the intestinal mucosal barrier by secreting multiple toxins, including α and β toxins, resulting in diarrhea, hemorrhage, and even mortality [3]. It imposes substantial economic losses on the global livestock industry [4,5]. Traditional control strategies for *C. perfringens* mainly rely on antibiotics [6], whose long-term use has been found to promote the emergence of antibiotic-resistant bacterial strains and to disrupt the balance of the intestinal microbiota, therefore increasing the risk of secondary infections. Robust resistance to high temperatures and chemical disinfectants is conferred by the spore-forming ability of *C. perfringens*, and full elimination through routine hygiene management is considered challenging [7]. Therefore, there is an urgent need to develop new, more targeted and eco-friendly control methods.

Bacteriophages (phages) are viruses that specifically target bacteria, and they represent a promising novel strategy for the control of *C. perfringens* [8,9]. Bacteriophages invade host bacterial cells by recognizing surface receptors. They replicate within the cells, and the progeny bacteriophages are released, eventually leading to the lysis of the bacteria [10]. Notably, the emergence of bacterial drug resistance is far less likely to be driven by phages [11]. To date, the vast majority of *C. perfringens* phages isolated from natural environments are classified within the class *Caudoviricetes* (a taxonomic group that now encompasses all tailed phages; the previously used families *Siphoviridae*, *Podoviridae*, and *Myoviridae* are no longer recognized under the current ICTV taxonomy) [12,13]. The genomes of these phages harbor genes that encode endolysins—enzymes that directly degrade the peptidoglycan layer of bacterial cell walls [14]. Importantly, these endolysins retain potent bactericidal activity even in the absence of the phage lytic cycle [15].

Growing evidence supports the broad-spectrum lytic activity of *C. perfringens* phages and their endolysins against multiple toxigenic strains, with stable activity under simulated intestinal conditions or simulated extreme conditions (pH 4–9, 4–50 °C), supporting their application in murine models [16]. The mouse model, a widely used system with well-established protocols, enables the systematic evaluation of phage efficacy, mechanisms, and safety. Phage vB_CpeP_15N3 has demonstrated efficacy in reducing *C. perfringens* loads and pathology in piglets via endolysin-mediated cell wall cleavage, and its stability suggests suitability for oral use in mice [17]. Although *C. perfringens* phage (CPP) research has advanced in poultry and piglets, mouse models remain critical for assessing host range, intestinal survival, and microbiota interactions, thereby providing precise experimental evidence to support innovative strategies for controlling *C. perfringens* infections in animal husbandry and human foodborne disease [16,18,19].

## 2. Materials and Methods

### 2.1. Materials

#### 2.1.1. Primary Reagents

Pancreatin–sulfite–cycloserine agar base (TSC), D-cycloserine solution, brain heart infusion broth (BHI), SM buffer, MRS agar, Trypticase Soy Broth (TSB), agar powder, oxygen indicators, anaerobic gas-generating bags, and sterile liquid paraffin were purchased from QingDao Hi-Tech Industrial Park Hope Bio-Technology Co., Ltd. (Qingdao, China).; viral genomic DNA/RNA extraction kits were obtained from Tiangen Biotech (Beijing) Co., Ltd. (Beijing, China). The TB Green^®^ Premix Ex Taq™ II kit was purchased from Takara Bio Inc. (Shiga, Japan). The FastPure Gel DNA Extraction Mini Kit was purchased from Nanjing Vazyme Biotech Co., Ltd. (Nanjing, China). Anaerobic jars were procured from Oxoid Limited (Basingstoke, UK), and PEG8000 was supplied by Beijing Coolaber Technology Co., Ltd. (Beijing, China).

#### 2.1.2. Source of Pathological Material and Experimental Animals

The experimental samples and animals included 15 sewage samples collected from a large-scale dairy farm in Hohhot, Inner Mongolia, China (used for phage isolation). Fifteen healthy SPF Kunming mice (6–8 weeks old, 16–20 g in weight) were purchased from SPF (Beijing) Biotechnology Co., Ltd (Beijing, China). All the mice were housed in the Laboratory Animal Center of the College of Veterinary Medicine, Inner Mongolia Agricultural University (Hohhot, China), with ad libitum access to food and water. The experimental procedures were conducted in strict accordance with the guidelines approved by the Institutional Animal Care and Use Committee of Inner Mongolia Agricultural University (approval number: NND2022023; approval date: 7 March 2022).

### 2.2. Methods

#### 2.2.1. Isolation and Purification of Bacteriophages

Phage isolation and purification were performed with partial optimizations based on the methods described by B. Liang et al. [20]. Sewage samples were centrifuged at 2000× *g* for 20 min, and the supernatant was mixed with SM buffer at a 1:1 volume ratio. Sewage-supplemented BHI medium was prepared by substituting purified water with the sewage–SM buffer mixture according to the standard BHI medium formulation. Twenty laboratory-preserved *C. perfringens* type A isolates were selected as host strains. These strains were inoculated into BHI medium and anaerobically cultured at 45 °C until the logarithmic growth phase (OD_600_ = 0.6) was reached. Enriched host cultures were obtained. A 100 μL aliquot of the enriched culture from each *C. perfringens* strain was inoculated into the sewage-supplemented BHI medium, and anaerobic incubation was performed at 37 °C for 16 to 24 h. The cultures were then harvested and centrifuged at 11,000× *g* and 4 °C for 20 min. The supernatant was collected and filtered through a 0.22 μm sterile filter.

BHI medium containing 0.6% agar was prepared as the overlay agar, and TSC medium was used as the bottom agar for double-layer agar plates. For the spot assay, 100 μL of logarithmic-phase *C. perfringens* suspension was mixed with the overlay agar, and the mixture was poured onto the solidified bottom agar. After the overlay agar solidified, 10 μL of the filtered supernatant was spotted onto each plate. Three replicates were set for each sewage sample. All plates were anaerobically incubated at 37 °C for 16 to 24 h, and plaque formation was observed.

For samples with visible plaques, the corresponding supernatant was subjected to serial ten-fold dilutions. A 100 μL aliquot of each dilution was mixed with 100 μL of logarithmic-phase *C. perfringens* suspension (1:1 volume ratio), and anaerobic incubation was performed at 37 °C for 30 min to allow phage adsorption. The mixture was then mixed with overlay agar and poured onto the bottom agar plate. After solidification, anaerobic incubation was performed at 37 °C for 16 to 24 h. Single, well-separated plaques were picked from the plates and transferred to 500 μL of SM buffer followed by overnight incubation at 4 °C. The next day, the suspension was centrifuged and the supernatant was collected for serial dilutions. Phage purification was repeated three to four times using the double-layer agar plate method. Clear, translucent, and uniformly sized plaques were obtained, and these were considered to indicate successful purification of the *C. perfringens* phage. The purified phage suspension was mixed with 20% glycerol at a 1:1 volume ratio and stored at −80 °C for long-term preservation.

#### 2.2.2. Concentration of Bacteriophages

The phage concentration was determined using the methods described by P. Li et al. [21] with further optimizations. Logarithmic-phase host *C. perfringens* cultures and the purified phage suspension were inoculated into 100 mL of BHI liquid medium at a multiplicity of infection (MOI) of 0.1. Anaerobic incubation was performed at 37 °C for 16 to 24 h for phage amplification. After incubation, the culture was centrifuged at 11,000× *g* and 4 °C for 10 min, and bacterial debris was removed. DNase I and RNase I were added to the collected supernatant to a final concentration of 1 µg/mL each. The mixture was incubated at room temperature (25 °C to 28 °C) for 30 min, and non-phage-derived nucleic acids were degraded. The treated supernatant was centrifuged again at 11,000× *g* for 20 min, and residual precipitates were removed. The supernatant was retained. An equal volume of 20% (*w*/*v*) PEG-8000 containing 1 M NaCl was added to the supernatant. Phage precipitation was carried out overnight at 4 °C. The mixture was centrifuged at 11,000× *g* and 4 °C for 20 min. The supernatant was discarded, and 3.0 mL of SM buffer was added to the phage pellet. The pellet was then vortexed vigorously for 15 s, and full resuspension was achieved. An equal volume of chloroform was added to the resuspended solution, and the mixture was mixed thoroughly by inversion. Centrifugation was performed at 2000× *g* and 4 °C for 20 min. The upper aqueous phase, which contained the phages, was carefully collected. This chloroform extraction step was repeated three to four times, and residual PEG and impurities were removed. The final collected supernatant was designated as the concentrated phage suspension and stored at 4 °C for short-term use.

#### 2.2.3. Transmission Electron Microscopy

Transmission electron microscopy (TEM) was used to observe phage morphology using a JEM-1400 instrument (JEOL Ltd., Tokyo, Japan). An aliquot (20 μL) of the purified and concentrated phage suspension was pipetted onto a copper mesh and allowed to adsorb naturally for 5–10 min. Excess liquid was gently blotted away with filter paper strips. After the residual liquid was completely absorbed, the mesh was slightly air-dried. Another 20 μL aliquot of 2% phosphotungstic acid (PTA) solution was then pipetted onto the same copper mesh and left to stand for 3–5 min for negative staining. Excess staining solution was removed with filter paper strips, and the copper mesh was dried under an incandescent lamp. The phage morphology was observed and imaged using the transmission electron microscope.

#### 2.2.4. Phage Titers

The phage titer was determined using the double-layer agar plate method. Purified phage suspension was subjected to 10-fold serial dilutions with SM buffer. A 100 μL aliquot of each dilution was mixed with an equal volume (100 μL) of logarithmic-phase host *C. perfringens* suspension, and the mixture was incubated in a 37 °C constant-temperature anaerobic incubator for 30 min to allow phage adsorption. Subsequently, 4 mL of 0.6% BHI agar medium (melted and cooled to 45 °C) was added to the mixture, vortexed thoroughly, and immediately poured onto pre-solidified TSC agar bottom plates. After the overlay agar solidified completely, the plates were inverted and incubated in a 37 °C constant-temperature anaerobic incubator for 16–24 h. The plaque morphology was observed, and the phage titer was calculated using the following formula: PFU/mL = Number of plaques × Dilution factor/Volume of diluted phage suspension added (mL). The detection limit now clearly defined as <1 PFU/mL. This corresponds to the lowest detectable titer using the plaque assay with the lowest dilution (undiluted sample) and 100 µL plating volume.

#### 2.2.5. Bacteriophage Lytic Spectrum

A laboratory-isolated *C. perfringens* type A strain was revived and expanded in culture, and a 200 μL aliquot of the host bacterial suspension (OD_600nm_ = 0.6) was added to 4 mL of 0.6% agar BHI semi-solid medium, mixed rapidly, and poured onto pre-solidified TSC agar bottom plates. After the overlay medium solidified, 10 μL of the purified phage suspension was spotted onto the plate surface, and the plates were incubated in a 37 °C constant-temperature anaerobic incubator for 16–24 h, with plaque formation used as the indicator to evaluate phage lytic activity against *C. perfringens* type A. For cross-lytic activity assessment, seven non-specific bacterial strains—*Escherichia coli*, *Streptococcus agalactiae*, *Pasteurella multocida*, *Staphylococcus aureus*, *Actinomyces* sp., *Salmonella* sp., and *Bifidobacterium* sp. (cultured in MRS medium)—were revived and expanded separately: the bottom layer medium for most non-specific strains was TSB containing 5% bovine serum and 1.5% agar, with the top layer medium being TSB semi-solid medium supplemented with 5% bovine serum and 0.6% agar. A 200 μL volume of the revived bacterial suspension of each non-target strain was thoroughly mixed with the corresponding top layer medium and poured onto the pre-solidified bottom layer medium, and after solidification, 10 μL of the purified phage suspension was spotted onto the plate surface, followed by incubation at 37 °C for 16–24 h, and cross-lytic activity was determined by observing plaque formation.

#### 2.2.6. Multiplicity of Infection (MOI)

A laboratory-isolated *C. perfringens* type A strain was revived and expanded in culture. The strain was then inoculated into BHI liquid medium and anaerobically cultured until the mid-logarithmic phase (OD_600_ = 0.6) was reached. A MOI gradient was established with values of 0.001, 0.01, 0.1, 1, 10, and 100. The phage suspension concentration was adjusted to match each MOI value. Equal volumes of the mid-logarithmic-phase host bacterial suspension and the phage suspension at each concentration were mixed thoroughly. Anaerobic incubation was performed at 37 °C for 16 to 20 h. After incubation, the mixtures were centrifuged at 11,000× *g* for 20 min, and the supernatants were collected. The phage titer in each supernatant was determined using the double-layer agar plate method. The MOI that corresponded to the highest phage titer was determined to be the optimal MOI for the phage.

#### 2.2.7. Thermal Stability Determination

A mid-logarithmic-phase *C. perfringens* type A culture (OD_600nm_ = 0.6) was used as the host, and the phage stock concentration was adjusted to the previously determined optimal MOI. Equal volumes of the host bacterial culture and the adjusted phage suspension were thoroughly mixed, and then aliquoted into sterile centrifuge tubes pre-filled with 5 mL of BHI liquid medium. Three replicate tubes were set up for each temperature (25 °C, 30 °C, 37 °C, 40 °C, 45 °C, and 50 °C), and all the tubes were sealed with sterile liquid paraffin to maintain anaerobic conditions. The tubes were incubated in constant-temperature anaerobic incubators at the respective temperatures for 16–20 h. After incubation, the cultures were centrifuged at 11,000× *g* for 20 min, and the supernatants were collected. The phage titer in each supernatant was determined using the double-layer agar plate method, and the collected data were analyzed to evaluate the effect of temperature on phage activity.

#### 2.2.8. pH Tolerance Test

BHI liquid medium was adjusted to pH values of 3, 4, 5, 6, 7, 8, 9, 10, 11, and 12, respectively. Sterile Eppendorf tubes were prepared, and 900 μL of sterile BHI liquid medium with each corresponding pH gradient was added to each tube. Subsequently, 100 μL of phage solution (10^10^ PFU/mL) was pipetted into each tube, mixed thoroughly, and incubated in a 37 °C constant-temperature incubator for 4 h. After incubation, the phage titer in each tube was determined using the double-layer agar plate method. Three parallel samples were set up for each pH gradient, and experimental data were recorded to analyze the effect of pH on phage activity.

#### 2.2.9. Growth Curve Analysis

Based on the method described by Wu [17] with modifications, bacteria were infected at a high MOI of 0.1. A 50 mL phage suspension (10^7^ PFU/mL) was mixed with 50 mL of *C. perfringens* type A culture (≈10^8^ CFU/mL, OD_600nm_ = 0.6). After thorough mixing and adsorption for 10 min at 37 °C, the mixture was centrifuged (5000× *g*, 1 min). The pellet was washed twice with SM buffer to remove unadsorbed phages, and then resuspended in fresh BHI medium. The resuspension was dispensed into sterile centrifuge tubes (3 mL/tube) for 9 time points (every 20 min for the first hour, and then every 30 min up to 4 h) with three biological replicates per time point. All tubes were incubated anaerobically at 37 °C. At each time point, one set of replicate tubes was collected (9 total samplings), and titers were determined immediately after chloroform treatment.

#### 2.2.10. Lysis Curve Determination

The phage suspension adjusted to the previously determined optimal MOI was mixed with an equal volume of mid-logarithmic-phase *C. perfringens* type A culture (OD_600nm_ = 0.6). The mixture was added to 50 mL of BHI liquid medium and mixed thoroughly, and then dispensed into sterile tubes (2 mL per tube) to set up 12 tubes corresponding to 12 time points. Simultaneously, bacterial control tubes (host bacteria without phage) and blank control tubes (BHI medium without bacteria or phage) were set up following the same specifications (12 tubes per control group, 2 mL per tube). All tubes were anaerobically incubated at 37 °C, and one tube from each group (phage- exposed, bacterial control, blank control) was collected every 2 h to measure the OD_600nm_ value (for a total of 12 sampling events). The experiment was performed in three independent biological replicates, and the collected data were recorded to plot the phage lysis curve (with bacterial growth inhibition reflected by OD_600nm_ changes).

#### 2.2.11. Whole-Genome Sequencing Analysis

Phage genomic DNA was extracted and isolated strictly according to the manufacturer’s instructions for the viral DNA extraction kit. The extracted DNA samples were sent to Sangon Biotech (Shanghai) Co., Ltd. (Shanghai, China) for whole-genome sequencing. All publicly available complete genome sequences of *C. perfringens* phages were retrieved from the NCBI database. A preliminary screening was performed based on genome length and integrity, and 22 reference genomes were obtained. The isolate vB_CppII_Hohhot was used as the query sequence. Similarity to the reference strains was analyzed via BLAST or FastANI (v1.33). Eleven reference strains were found to have more than 95% similarity, and the remaining 11 strains exhibited 70% to 95% similarity. Multiple sequence alignment of whole genomes or core genes was performed using MAFFT (version 7.526). A phylogenetic tree was constructed with MEGA-X (version X) software [22] under the maximum likelihood method, and 1000 bootstrap replicates were applied. FastANI was used to calculate the average nucleotide identity (ANI) between the isolate and all reference genomes. Genome integrity assessment and functional annotation were conducted using phage-specific analysis platforms (PhageScope [23], NCBI [24], NR [25], COG [26], CO [27], CDD [28]) and SnapGene (version 8.2.2) software (for gene mapping). Antibiotic resistance gene annotation was performed via the Comprehensive Antibiotic Resistance Database (CARD) [29] (https://card.mcmaster.ca, accessed on 29 August 2025) and the Center for Genomic Epidemiology platform (https://www.genomicepidemiology.org/services/, accessed on 29 August 2025). Virulence gene annotation and virulence factor classification were completed using the Virulence Factor Database (VFDB) [30] (http://www.mgc.ac.cn, accessed on 29 August 2025).

#### 2.2.12. Prophylactic Evaluation of Phage vB_CppII_Hohhot in a Mouse Model

Briefly, *C. perfringens* type A (CPA) strain was inoculated onto TSC agar plates, and a single colony was then inoculated into 4 mL of BHI medium, followed by anaerobic incubation at 45 °C for 16–24 h. After determination of the bacterial titer, the suspension was centrifuged at 2000× *g* for 20 min. The bacterial pellet was resuspended in sterile physiological saline and adjusted to a final concentration of 10^8^ CFU/mL for subsequent use. Laboratory-frozen CPP was thawed and adjusted to a titer of 10^8^ PFU/mL. Then, 100 μL of *C. perfringens* suspension and 100 μL of CPP suspension were mixed, added to 8 mL of BHI medium, and incubated overnight, and the resulting lysate was harvested for later use.

Fifteen healthy Kunming mice were randomly divided into three groups (n = 5 per group): the challenge group was intraperitoneally injected with 300 μL of the prepared *C. perfringens* suspension (10^8^ CFU/mL). The in vitro lysis group was intraperitoneally injected with 300 μL of the unprocessed lysate mixture of *C. perfringens* and CPP (*C. perfringens* titer 10^8^ CFU/mL, CPP titer 10^8^ PFU/mL). The blank control group was intraperitoneally injected with 300 μL of sterile PBS buffer. The general condition of the mice was continuously monitored for 7 days, and mortality was recorded daily. At the end of the experiment, all mice were euthanized and subjected to necropsy. Tissue samples of the heart, liver, lung, spleen, kidney, duodenum, jejunum, and ileum were collected from each mouse. One part of the samples was processed for histopathological sectioning to observe morphological changes under light microscopy, while the other part was analyzed by qPCR to determine the relative expression levels of alpha-toxin, allowing the evaluation of significant differences between each organ sample and the control group.

#### 2.2.13. Pathological Section Analysis

For histological and pathological analysis, the tissues were processed and sectioned as previously described by Biancardi et al. [31,32,33]. The samples were rapidly dissected and trimmed into ~5 mm × 5 mm × 2 mm tissue blocks. To prevent atelectasis, lungs were tracheally perfused with 10% neutral buffered formalin (NBF). All tissues were fixed in 10% NBF at room temperature for 24–48 h (tissue-to-fixative ratio 1:10–1:20), and then transferred to 70% ethanol for storage at 4 °C. Tissue dehydration was performed using a graded ethanol series (70% for 12 h, 80% for 2 h, 90% for 2 h, 95% for 1 h, 100% for 1 h). Following xylene clearing, tissues underwent gradient paraffin infiltration at 60–62 °C and were embedded in molten paraffin to form blocks. Serial sections of 4–5 μm thickness were cut using a rotary microtome, and mounted in 45 °C warm water, transferred to poly-L-lysine-coated slides, and baked at 60 °C for 45–60 min. For hematoxylin–eosin (H&E) staining, sections were dewaxed with xylene, rehydrated, stained with Harris’ hematoxylin (3–8 min), differentiated with 1% hydrochloric acid–alcohol, and counterstained with 0.5% eosin Y (1–3 min). Finally, sections were dehydrated, cleared with xylene, and mounted. All sections were examined under a light microscope for tissue structure, cell morphology, and pathological changes, with photographic documentation.

#### 2.2.14. SYBR Green qPCR Analysis

**Figure 1:**
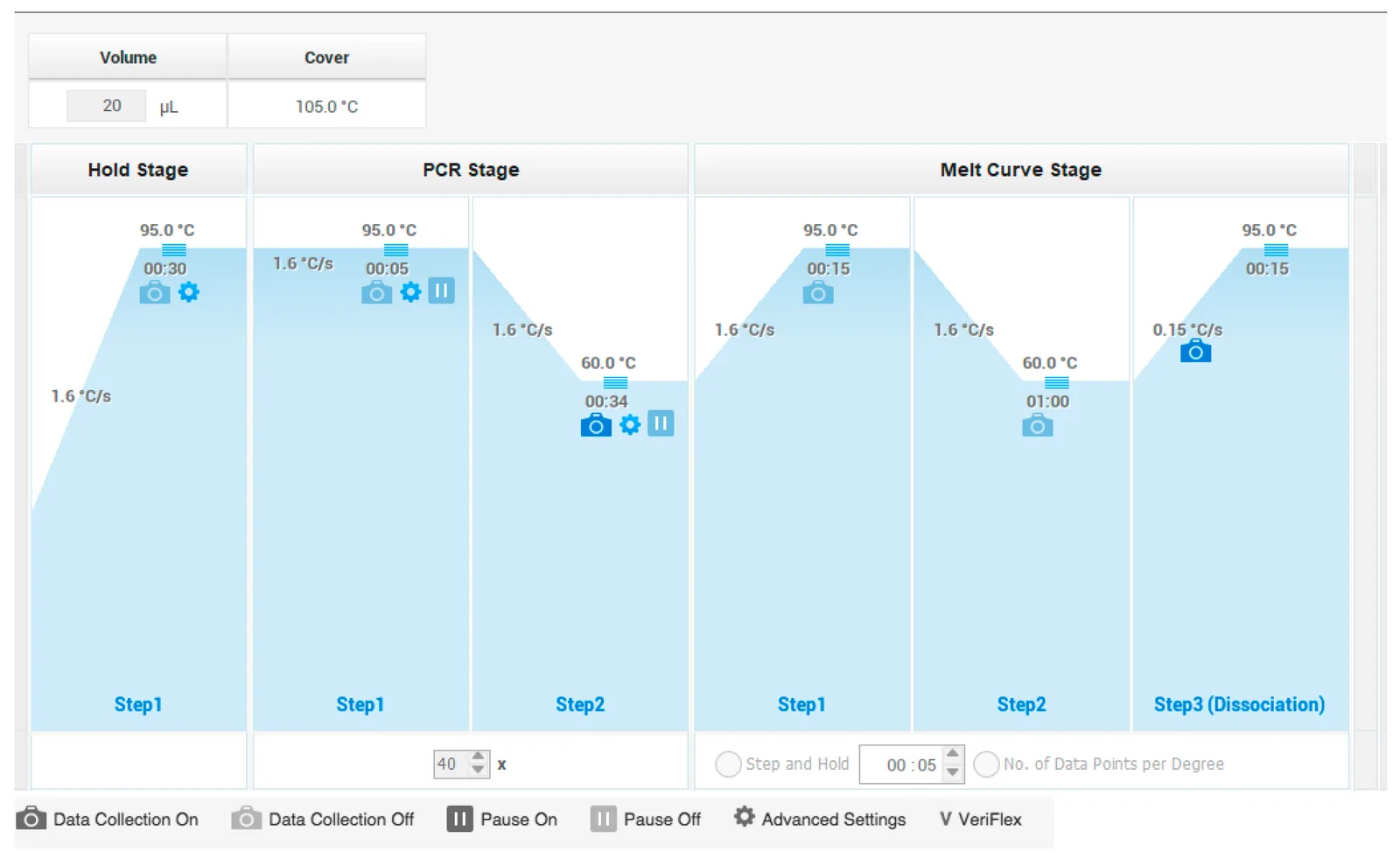
qPCR thermal cycling conditions for detection of the *C. perfringens* alpha toxin gene (plc).

To investigate the significance of the differences between CPP and *C. perfringens*, we employed the SYBR Green qPCR method. Mouse organs were homogenized in 1 mL of BHI liquid medium. DNA was extracted from the homogenate strictly following the protocol of the bacterial DNA extraction kit. The concentration (ng/μL) of the extracted DNA was measured using a NanoDrop 2000 spectrophotometer (Thermo Fisher Scientific, Wilmington, DE, USA), and the DNA samples were diluted to below 100 ng/μL for subsequent use. Primers for α-toxin (CPA-F: 5′-CTTATTTGTGCCGCGCTAGC-3′; CPA-R: 5′-CCTAATTGAAGCTCATGCATGTTC-3′) were designed in-house. β-Actin primers were referenced from JIANG et al. [34] (F: 5′-CTAGGCGGACTGTTACTGAGC-3′; R: 5′-CGCCTTCACCGTTCCAGTTT-3′) and synthesized by Sangon Biotech (Shanghai) Co., Ltd. The PCR mixture was prepared on ice strictly according to the instructions of the TB Green^®^ Premix Ex Taq™ II Reagent Kit. The total reaction volume was 20 μL, consisting of 10 μL of TB Green Premix, 0.8 μL each of the forward and reverse primers, 0.4 μL of ROX Reference Dye II, 2 μL of DNA template, and 6 μL of ddH_2_O. The PCR program is shown in Figure 1. After amplification with a PCR instrument, the data were organized and analyzed using Excel, and significance analysis plots were generated using GraphPad Prism 10 [35].

## 3. Results

### 3.1. Isolation and Titer Determination of Bacteriophages

**Figure 2:**
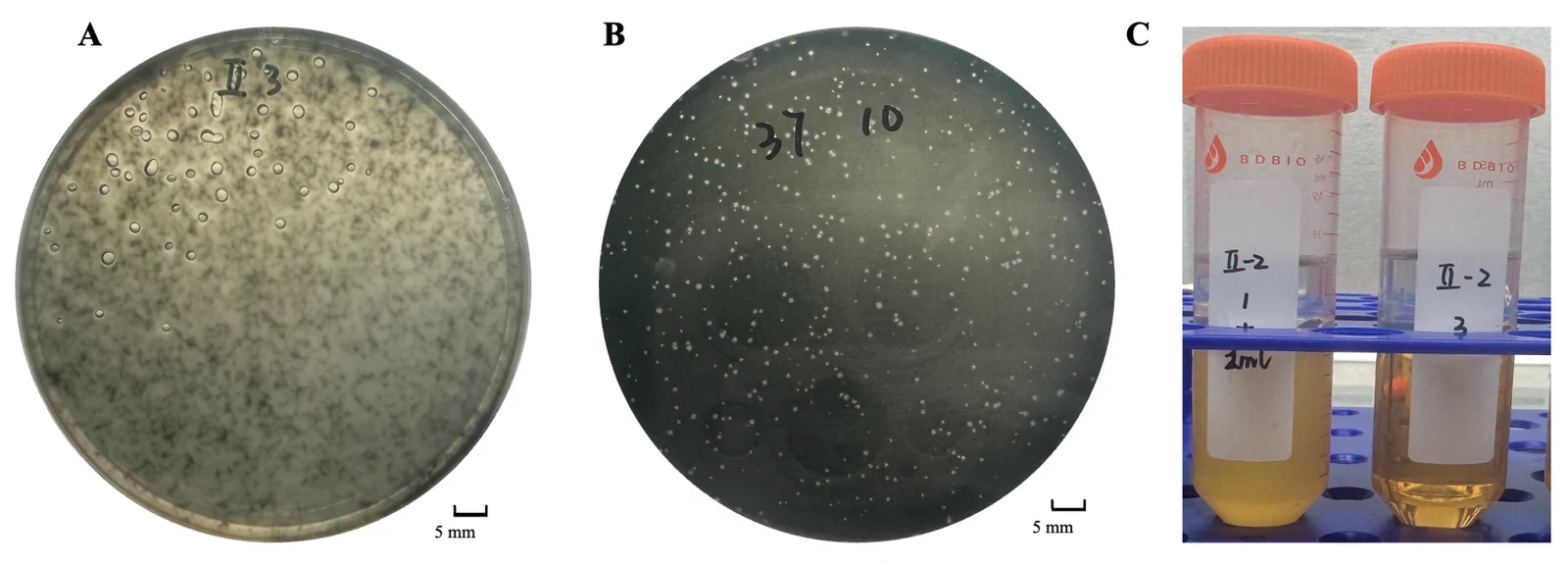
Isolation and lytic activity of Phage vB_CppII_Hohhot: (**A**) Phage lysis of *C. perfringens*. (**B**) Clear, translucent plaques were visible after purification. (**C**) Isolated phage causes the bacterial suspension to become transparent.

To isolate *C. perfringens* phages, the drop-inoculation method was used. A suspected *C. perfringens* phage was initially isolated. To amplify this suspected phage, anaerobic co-cultivation with its host bacteria was performed in brain heart infusion (BHI) liquid medium at a constant temperature. Subsequent characterization indicated that this isolate was a lytic bacteriophage, and complete lysis of its host strain was achieved. Multiple lines of evidence were used to verify the lytic activity of the phage (Figure 2). After 16 h of incubation, a phage titer of 3 (log_10_ PFU/mL) was reached. At this titer, nearly complete lysis of the host bacteria was observed (Figure 2A). Under the same incubation conditions, a phage titer of 10 (log_10_ PFU/mL) was reached. Distinct and clear plaques were formed by the phages (Figure 2B). When the phages were co-incubated with the host bacterium C. perfringens for 16 h, complete clearing of the bacterial culture was observed (Figure 2C).

The phage titer was quantified using the double-layer agar plate assay, and determined to be 1.96 × 10^14^ plaque-forming units (PFU)/mL. According to the nomenclature guidelines established by the International Committee on Taxonomy of Viruses (ICTV), the newly isolated pathogenic *C. perfringens* bacteriophage was designated as vB_CppII_Hohhot and deposited at the China General Microbiological Culture Collection Center (CGMCC, No. 46656).

### 3.2. Morphological Characterization

**Figure 3:**
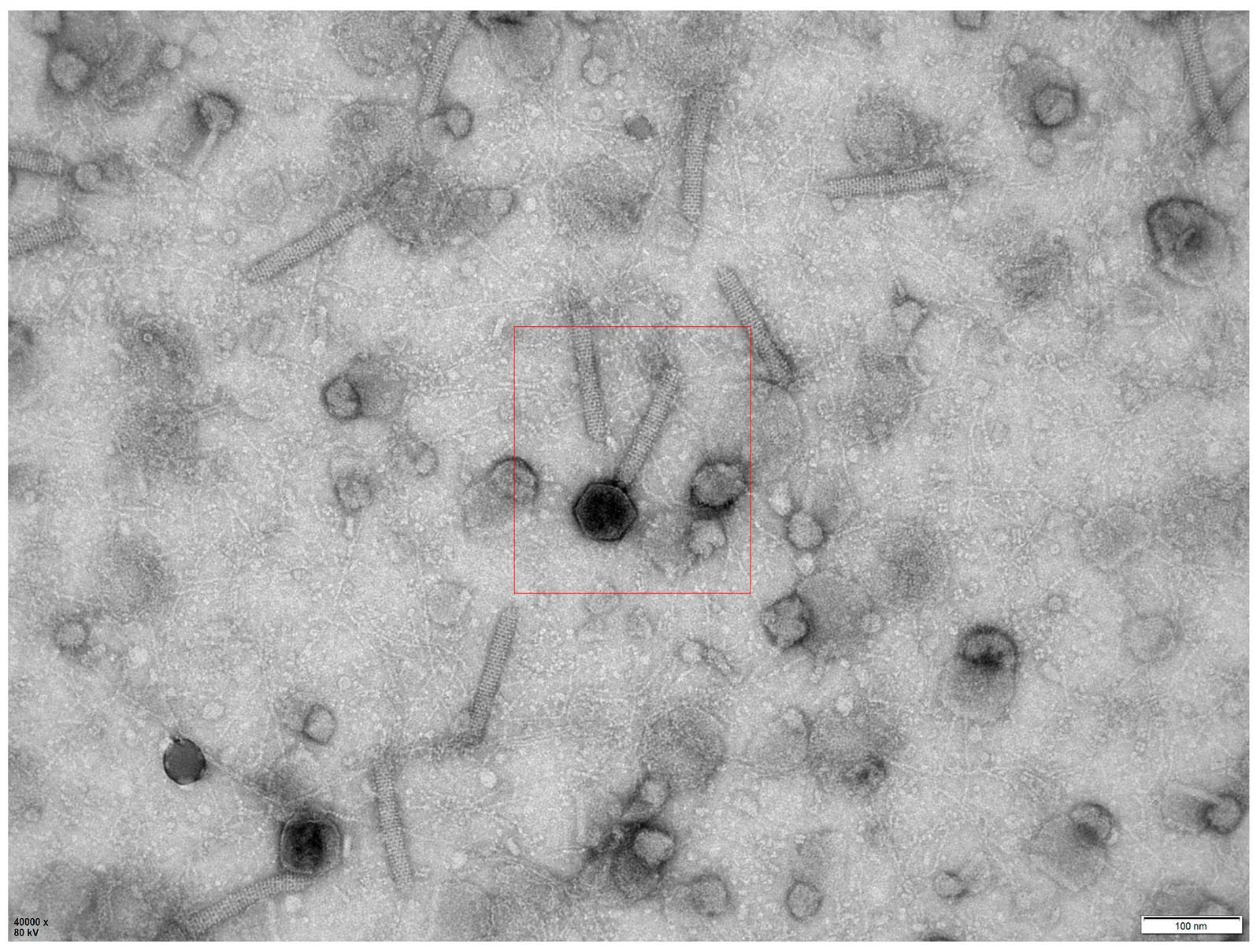
The morphology of phage vB_CppII_Hohhot under transmission electron microscopy (40,000× magnification, 80 kV).

To investigate the morphological features of the isolated bacteriophage, observation was performed under transmission electron microscopy (TEM). The results revealed that the bacteriophage vB_CppII_Hohhot possessed an icosahedral head and a helical tail, with a total length of approximately 201.94 nm (Figure 2). Specifically, the head had a maximum and minimum diameter of 66.88 nm and approximately 59.85 nm, respectively, while the tail had a length of 134.17 nm. These morphological characteristics were consistent with its classification into the class *Caudoviricetes* (Figure 3).

### 3.3. Determination of Phage Lytic Spectrum

**Figure 4:**
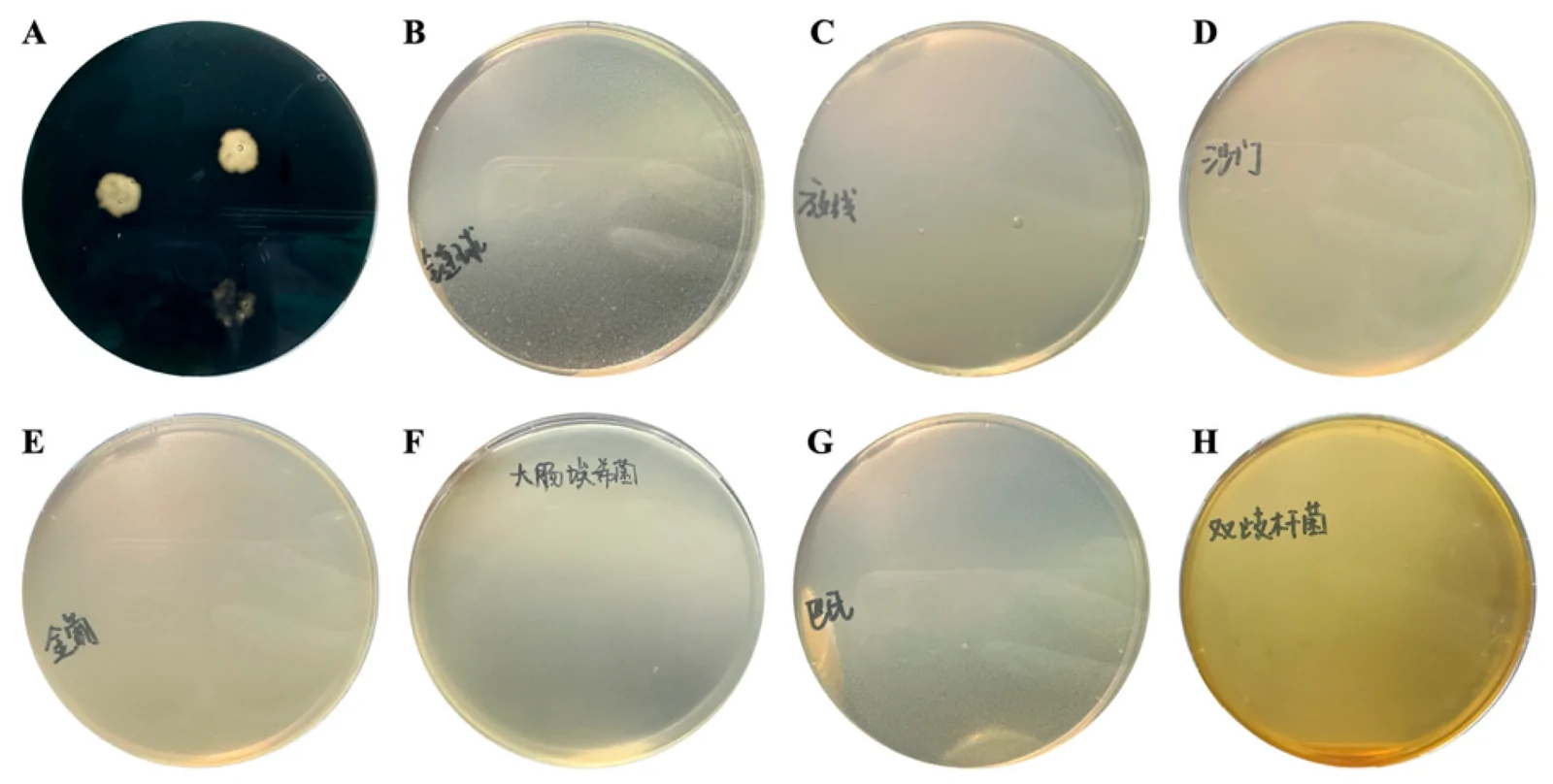
Lysis profile of the phage vB_CppII_Hohhot: (**A**) Clear lytic plaques are visible on the lawn of *C. perfringens*. (**B**–**H**) No lytic plaques were observed for the following species: *Streptococcus pneumoniae* (**B**), *Pseudomonas aeruginosa* (**C**), *Salmonella* spp. (**D**), *Staphylococcus aureus* (**E**), *Escherichia coli* (**F**), *Pasteurella multocida* (**G**), and *Bifidobacterium* spp. (**H**).

The plaque formation assay was used to assess the phage lysis spectrum. Lytic activity of phage vB_CppII_Hohhot against *C. perfringens* type A was observed (Figure 4A). No lytic activity was detected when the phage was tested against other bacterial strains. These strains included *Streptococcus pneumoniae* (Figure 4B), *Pseudomonas aeruginosa* (Figure 4C), *Salmonella* spp. (Figure 4D), *Staphylococcus aureus* (Figure 4E), *Escherichia coli* (Figure 4F), *Pasteurella multocida* (Figure 4G), and *Bifidobacterium* spp. (Figure 4H).

### 3.4. Biological Characteristics

**Figure 5:**
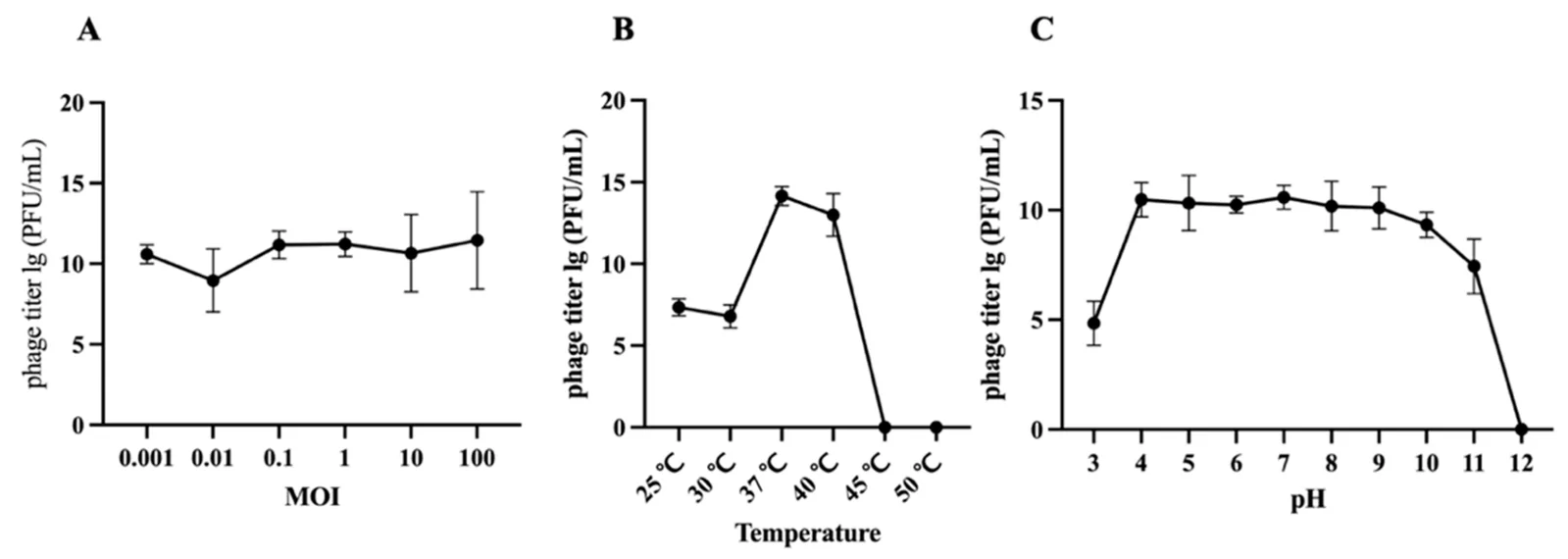
Biological characterization of phage vB_CppII_Hohhot. (**A**) Multiplicity of infection (MOI) assay; (**B**) thermal stability at different temperatures; (**C**) pH stability. All experiments were performed in triplicate, and data are presented as mean ± SD (n = 3). Data shown are from parameter-screening experiments; no statistical comparisons were made between groups.

The key biological characteristics of the lytic phage vB_CppII_Hohhot were detected in systematic assays based on data from laboratory experiments. An optimal MOI of 0.1 was determined for the phage, and the maximum titer was achieved at this relatively low phage dosage (Figure 5A). Temperature adaptability was assessed. The highest titer was observed when the phage was cultured at 37°C (10^14^ PFU/mL), and the titer fell below the detection limit at temperatures above 45°C (Figure 5B). pH tolerance was also assessed. A detectable titer was maintained at pH 3 (10^4.8^ PFU/mL). Stable titers were observed across the pH range of 4 to 11, but no viable phage was detected at pH 12 (detection limit: <1 PFU/mL) (Figure 5C).

### 3.5. Growth Kinetics and Host Lytic Capacity

**Figure 6:**
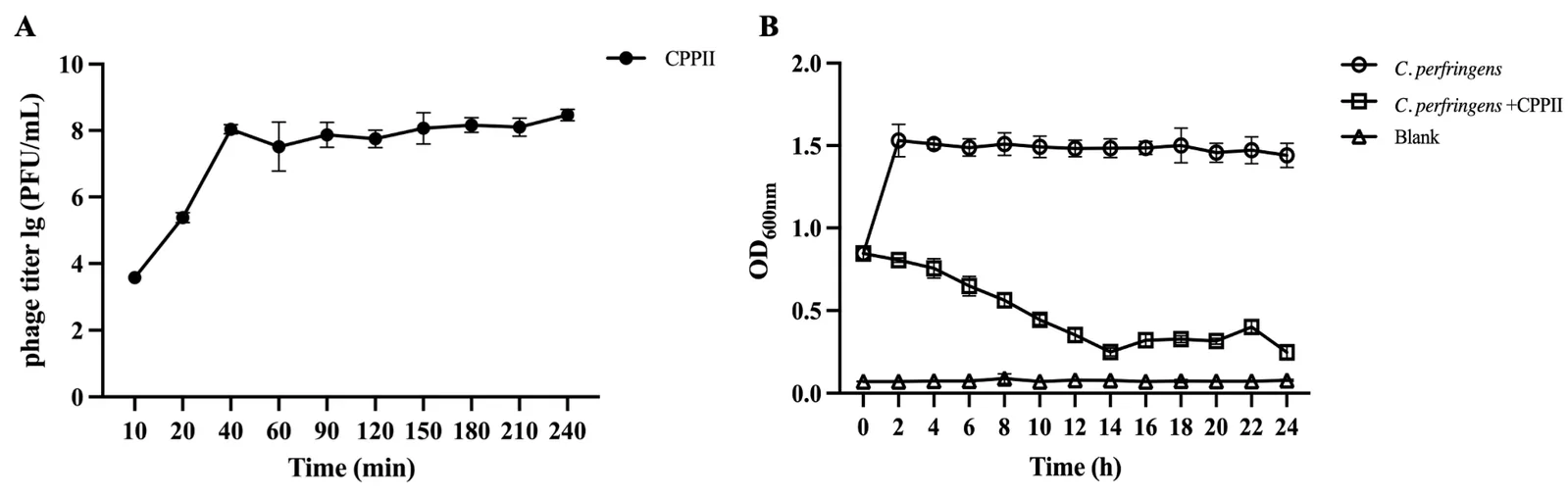
The growth curve and lysis curve of the phage vB_CppII_Hohhot: (**A**) The one-step growth curve of CPP. (**B**) The OD_600nm_ trends over 24 h for the *C. perfringens* host strain, the *C. perfringens* + CPP mixture, and the blank control.

The phage was co-cultured with its host *C. perfringens* at an optimal MOI of 0.1. Samples were taken every 20 min during the first hour, and then every 30 min thereafter for titer determination. Rapid phage proliferation was observed between 10 and 40 min, reaching a plateau at approximately 40 min. The eclipse period was approximately 10 min, the latent period approximately 40 min, and the burst size approximately 14.3 PFU/cell (Figure 6A). A parallel co-culture experiment was performed under identical optimal MOI conditions. Samples were harvested at 2 h intervals to assess host lysis. A significant reduction in bacterial density (OD_600nm_ decreased from 0.8 to 0.2–0.3, representing a 60–75% reduction) of the host *C. perfringens* were observed by 14 h of incubation, and lytic activity of the phage against the target strain was observed (Figure 6B).

### 3.6. Whole-Genome Sequencing and Annotation

Whole-genome sequencing of the phage vB_CppII_Hohhot was performed. The phage was found to have a full-length genome of 53,225 bp with a G + C content of 34.14%. Seventy-six predicted protein-coding genes (open reading frames, ORFs) were identified, ranging in length from 141 bp to 2562 bp. The total coding length was 47,457 bp, and the coding density was 86.16%. This profile was consistent with that of many known *C. perfringens* phages (CGMCC, No. 46656).

**Figure 7:**
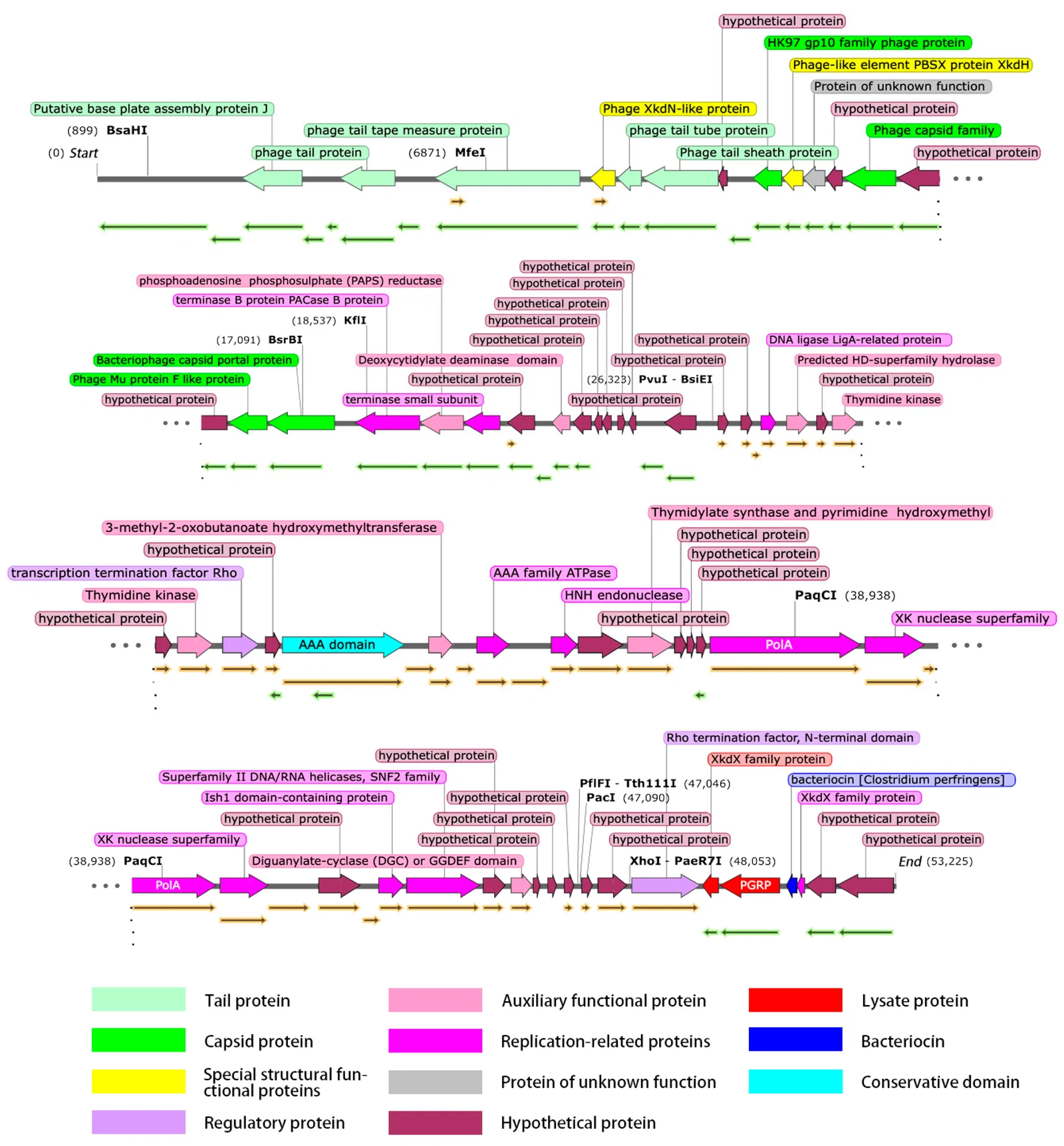
Genetic map of bacteriophage vB_CppII_Hohhot.

Functional annotation was performed on the 76 identified ORFs based on the whole-genome sequencing data. The amino acid sequence of each ORF was aligned against the NCBI BLASTp, NR, and CDD databases. Functional assignment was performed by referencing the protein sequences with the highest homology. A gene map was constructed via SnapGene software, and the spatial distribution of these ORFs across the genome was visualized (Figure 7). Sequence alignment indicated that 37 ORF-encoded products shared significant homology with proteins of known functions. The remaining 39 gene products had no matches with any viral or prokaryotic sequences in public databases.

Screening against the Comprehensive Antibiotic Resistance Database (CARD) was performed, and no known antibiotic resistance genes were identified in the genome. Analysis via the Virulence Factor Database (VFDB) initially identified *gene 72* as containing enterotoxin-related sequences. However, subsequent BLAST homology verification and conserved domain analysis using NCBI reannotated this gene as encoding an N-acetyl-cytidyl-L-alanyl aminopeptidase, a key endolysin that mediates host bacterial lysis. This reannotation was supported by the genomic context of *gene 72*, which is adjacent to other lysis-associated genes. A specific search for lysogeny-related genes was conducted. These genes included integrases (site-specific recombinases), tyrosine recombinases, serine recombinases, excisionases, and repressor proteins (e.g., Cro, CI). Searches were performed using PhageAI, PhageTyper, and manual BLASTP searches against the NCBI non-redundant database and ACLAME (a database of mobile genetic elements). No homologs of known lysogeny-associated genes were found. Integrase, recombinase, and repressor genes were absent. Typical lytic cycle genes, including holin, endolysin, and genes involved in DNA replication and packaging, were present.

### 3.7. Construction of Genetic Phylogenetic Tree

**Figure 8:**
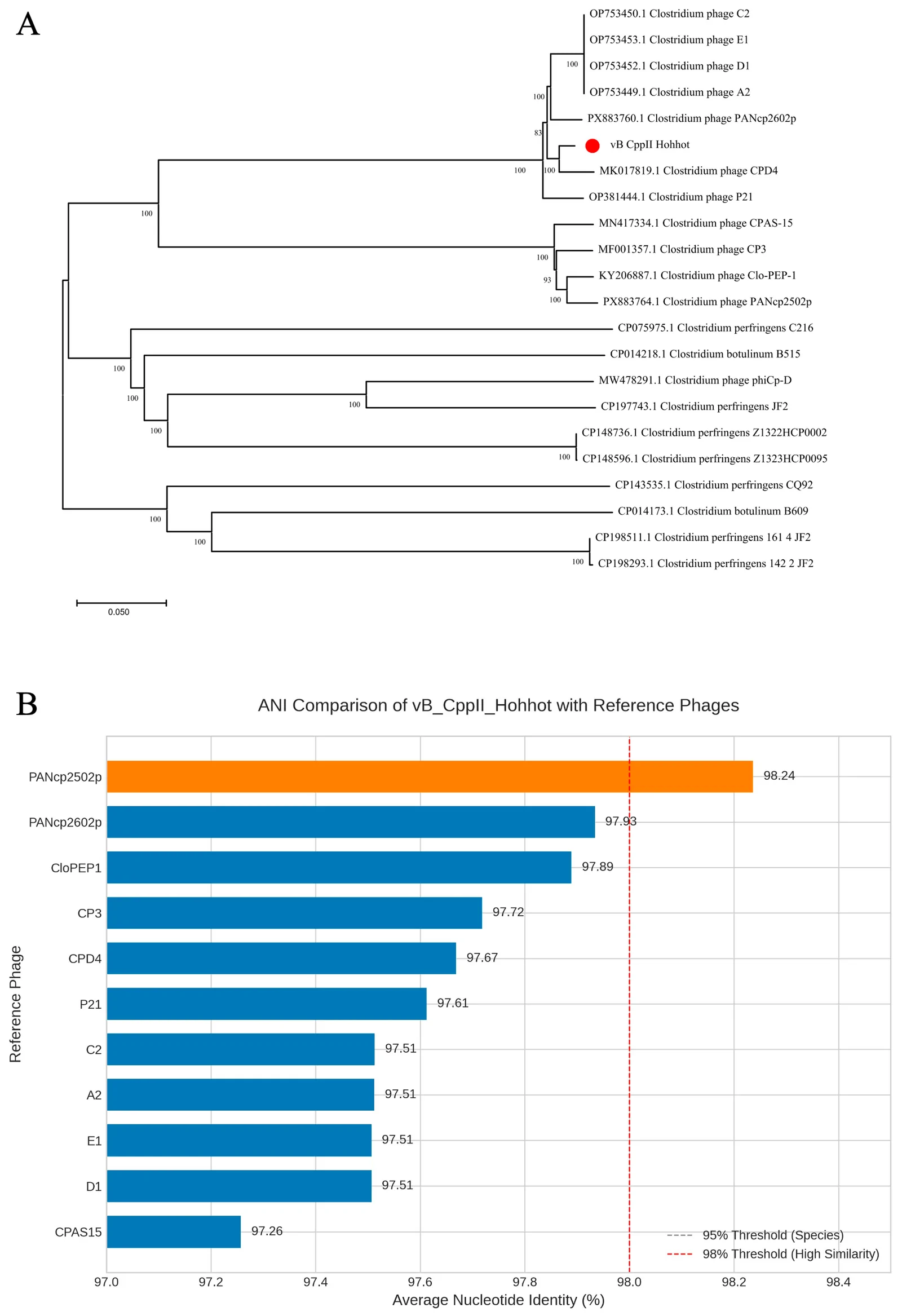
Phage gene evolutionary tree and ANI analysis. (**A**) Phylogenetic tree of vB_CppII_Hohhot and related phages based on whole-genome sequences; (**B**) Pairwise ANI values between vB_CppII_Hohhot and reference phages.

A phylogenetic tree was built with MEGA-X using the maximum likelihood method. BLAST results showed 97% whole-genome similarity between isolate vB_CppII_Hohhot and Clostridium phage CPD4. The isolate formed a well-supported clade with CPD4 and other Clostridium phages (Figure 8A). FastANI analysis gave ANI values of 97.2–98.2% against 11 known Clostridium phages (Figure 8B), with nearly full coverage. Consequently, vB_CppII_Hohhot was identified as a newly isolated within the existing species, distinct from previously characterized strains.

### 3.8. C. perfringens Challenge and Prophylactic Model

**Figure 9:**
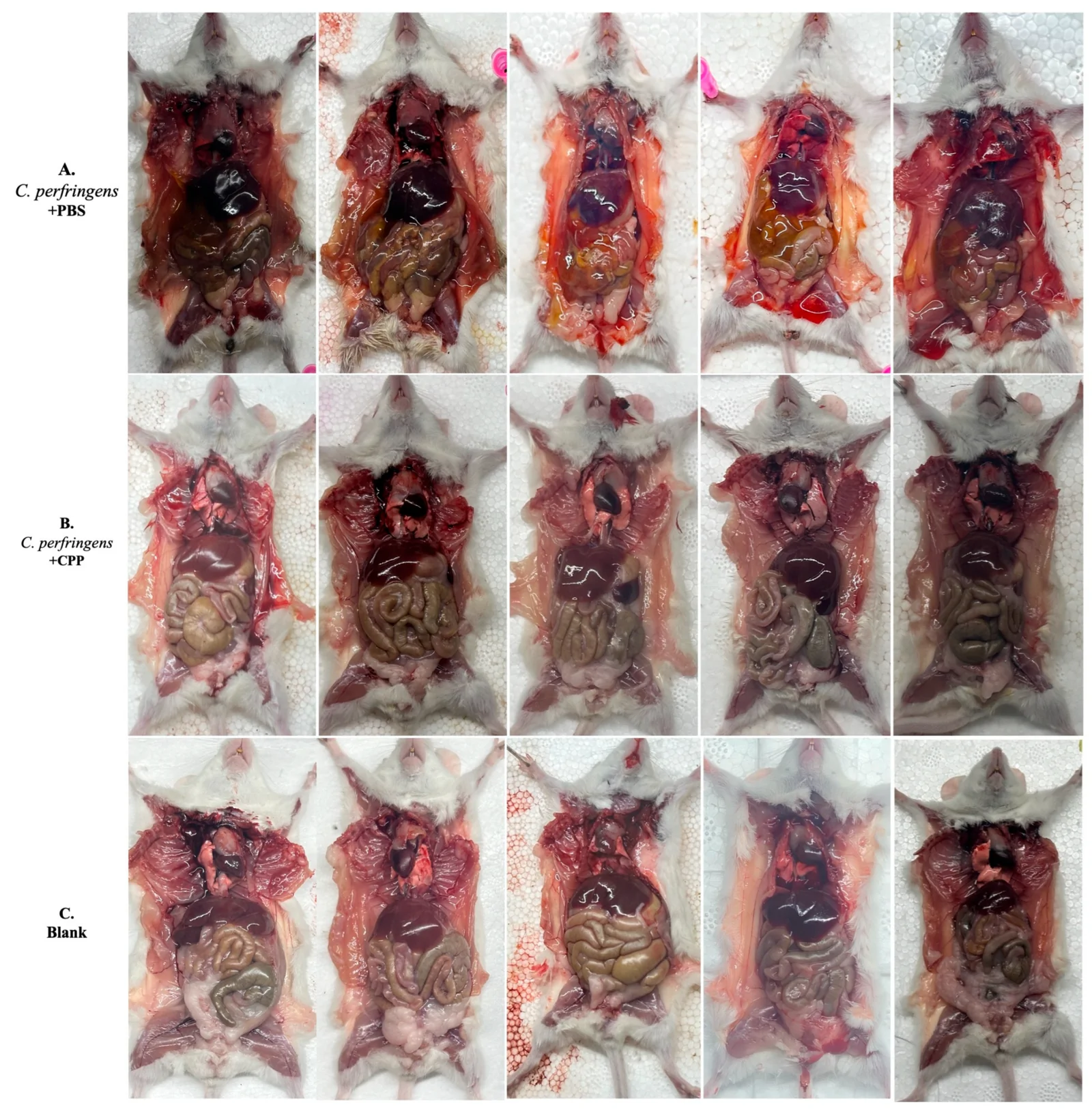
Necropsy on mouse model: Anatomical analysis of the mouse infection model. (**A**) Supine cross-sectional views of mice from the *C. perfringens* + PBS challenge group, (**B**) *C. perfringens* + CPP lysate group, and (**C**) blank control group at 7 days post-challenge.

To further investigate the preventive and therapeutic efficacy of vB_CppII_Hohhot against diseases caused by *C. perfringens* infection, experiments were conducted using mice as the animal model. Three experimental groups were established, including the *C. perfringens* + PBS challenge group, the *C. perfringens* + CPP lysate group, and the blank control group (PBS only). All mice in the *C. perfringens* + PBS challenge group succumbed to infection, whereas all mice in the other two groups survived. Necropsy of the deceased mice in the *C. perfringens* + PBS challenge group revealed hemorrhagic lesions in the heart, liver, spleen, and kidneys; gas gangrene in the intestines; and scattered hemorrhagic foci in the lungs, and these findings were consistent with the pathogenic characteristics of *C. perfringens* type A (Figure 9A). In contrast, necropsy of the surviving mice in the *C. perfringens* + CPP lysate group and the blank control group showed no significant pathological abnormalities in these organs (Figure 9B,C).

**Figure 10:**
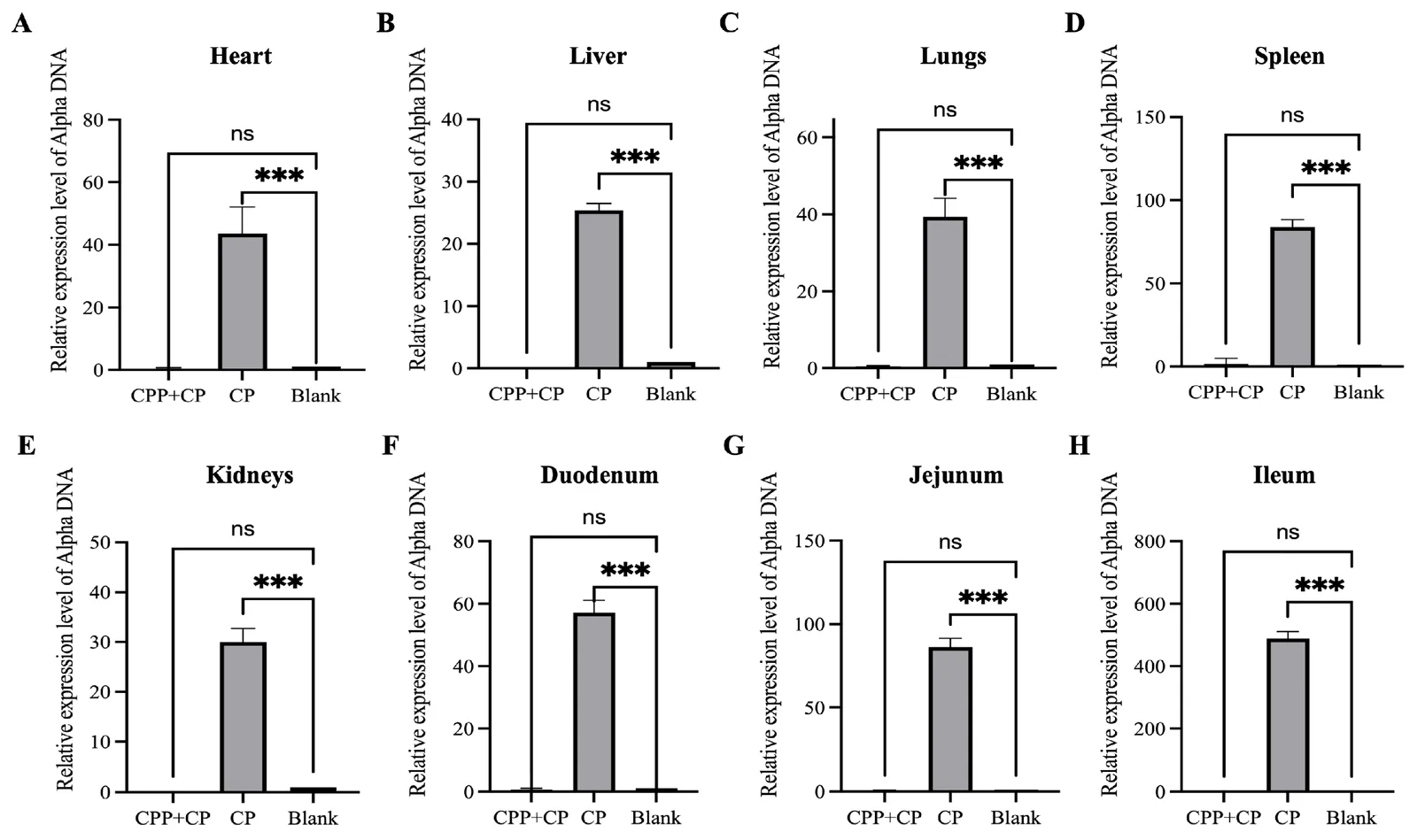
qPCR verification analysis on mouse model: (**A**–**H**) Analysis of the relative expression level of alpha toxin. qPCR analysis of α-toxin’s relative expression in the heart, liver, lungs, spleen, kidneys, duodenum, jejunum, and ileum. Data are normalized to the blank control group and presented as mean ± SD (n = 3). *** *p* < 0.001; ns, not significant.

DNA was extracted from tissue homogenates of the three groups for qPCR testing. Experimental data were organized and summarized using Excel. All statistical analyses were carried out with GraphPad Prism 10. Comparisons between three groups were performed using unpaired two-tailed Student’s *t*-test, while multiple group comparisons were assessed by one-way ANOVA followed by Tukey’s multiple comparisons test. Relative α-toxin levels in the heart, liver, lung, spleen, kidney, duodenum, jejunum, and cecum of mice in the three groups are shown in Figure 10A–H. No significant differences in α-toxin levels were observed in any organ between the *C. perfringens* + CPP group and the blank control group. In contrast, the α-toxin levels in the *C. perfringens* + PBS challenge group were significantly higher than those in the blank control group and the *C. perfringens* + CPP group.

**Figure 11:**
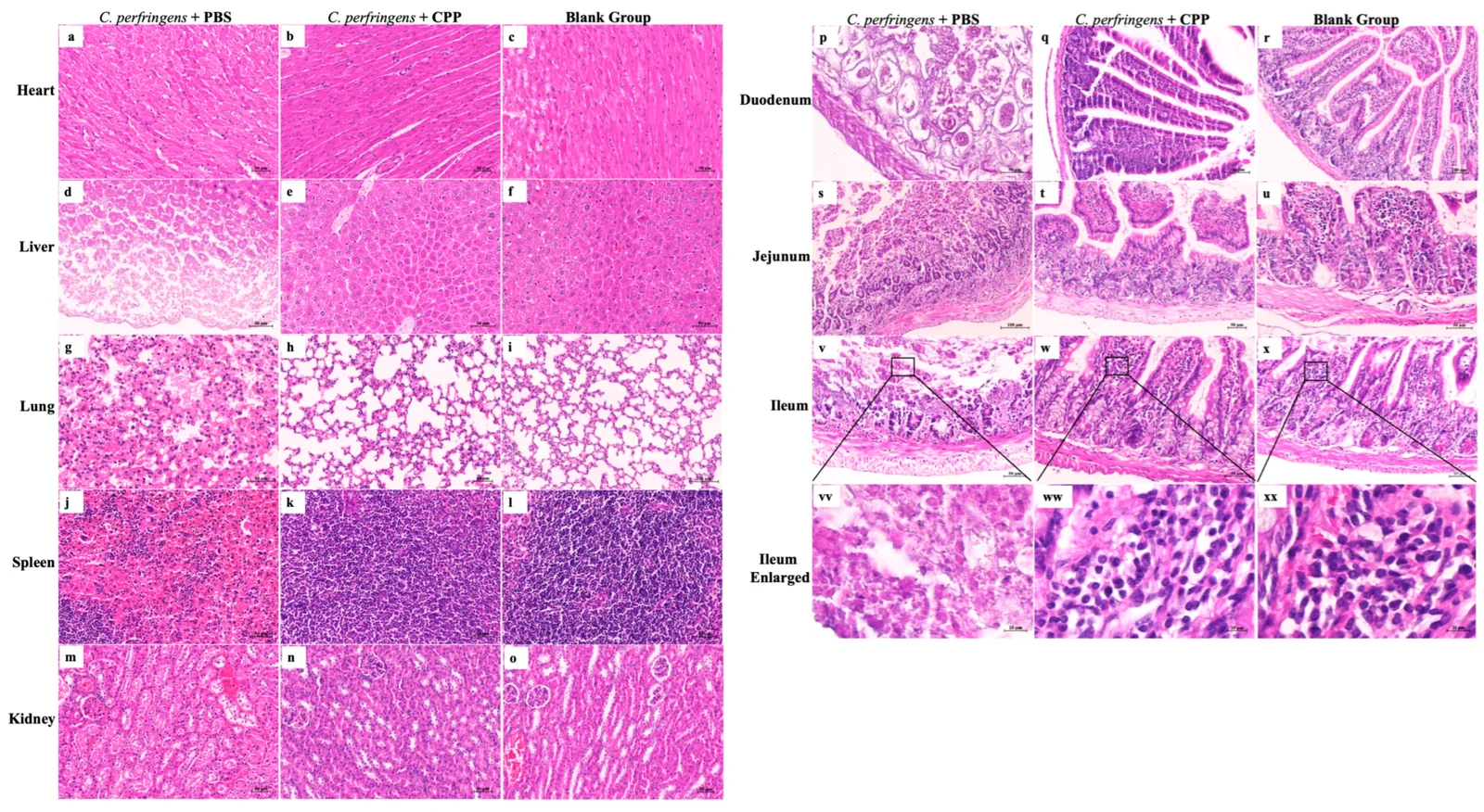
Histopathological evaluation of the prophylactic effect of phage vB_CppII_Hohhot in a mouse model. H&E-stained sections of heart, liver, lung, spleen, kidney, duodenum, jejunum, and ileum (scale bar, 10–100 μm). Pathological changes were assessed to evaluate tissue protection following phage pre-exposure. (**a**–**c**) Heart: (**a**) *C. perfringens* + PBS, (**b**) *C. perfringens* + CPP, (**c**) blank control. (**d**–**f**) Liver: (**d**) *C. perfringens* + PBS, (**e**) *C. perfringens* + CPP, (**f**) blank control. (**g**–**i**) Lung: (**g**) *C. perfringens* + PBS, (**h**) *C. perfringens* + CPP, (**i**) blank control. (**j**–**l**) Spleen: (**j**) *C. perfringens* + PBS, (**k**) *C. perfringens* + CPP, (**l**) blank control. (**m**–**o**) Kidney: (**m**) *C. perfringens* + PBS, (**n**) *C. perfringens* + CPP, (**o**) blank control. (**p**–**r**) Duodenum: (**p**) *C. perfringens* + PBS, (**q**) *C. perfringens* + CPP, (**r**) blank control. (**s**–**u**) Jejunum: (**s**) *C. perfringens* + PBS, (**t**) *C. perfringens* + CPP, (**u**) blank control. (**v**–**x**) Ileum: (**v**) *C. perfringens* + PBS, (**w**) *C. perfringens* + CPP, (**x**) blank control. (**vv**–**xx**) Ileum enlarged.

H&E staining was performed on sections of the heart, liver, lungs, spleen, and kidneys from all three experimental groups. In the heart (Figure 11a), myocardial fibers in the *C. perfringens* + PBS challenge group showed granular and hydropic degeneration. Some fibers displayed dissolution and fragmentation. No obvious pathological lesions were observed in the *C. perfringens* + CPP lysate group or the blank control group (Figure 11b,c).

In the liver, extensive necrotic foci were observed in the peripheral region of the *C. perfringens* + PBS challenge group. Hepatocytes within these foci showed signs of necrosis, and a clear boundary was observed between necrotic and normal areas. Marginal hepatocellular necrosis was found, characterized mainly by pyknosis, and no obvious inflammatory exudation was observed (Figure 11d). No obvious pathological lesions were observed in the *C. perfringens* + CPP lysate group or the blank control group (Figure 11e,f).

In the lungs, the alveolar septa were widened in the *C. perfringens* + PBS challenge group, and significant inflammatory exudation (mainly neutrophils) was observed. Fibrin exudation was found in some alveoli, along with occasional hemorrhagic foci. The alveolar spaces were filled with numerous red blood cells (Figure 11g). No obvious pathological lesions were observed in the *C. perfringens* + CPP lysate group or the blank control group (Figure 11h,i).

In the spleen, the volume of the white pulp was greatly reduced in the *C. perfringens* + PBS challenge group, but the volume of the red pulp was increased. The boundary between the red pulp and the white pulp was unclear in some areas, and these areas were filled with numerous red blood cells. The white pulp retained only the central artery, and almost complete depletion of lymphocytes was observed (Figure 11j). No obvious pathological lesions were observed in the *C. perfringens* + CPP lysate group or the blank control group (Figure 11k,l).

In the kidneys, renal tubular epithelial cells in the cortical region generally showed granular and hydropic degeneration in the *C. perfringens* + PBS challenge group. Some cells underwent necrosis, detached from the basement membrane, and exhibited pyknosis (Figure 11m). No obvious pathological lesions were observed in the *C. perfringens* + CPP lysate group or the blank control group (Figure 11n,o).

In the duodenum, all tissue cells in the mucosal, submucosal, muscular, and serosal layers became necrotic in the *C. perfringens* + PBS challenge group. Complete loss of structural architecture was observed. Severe necrotizing enteritis was observed (Figure 11p). No obvious pathological lesions were observed in the *C. perfringens* + CPP lysate group or the blank control group (Figure 11q,r).

In the jejunum, intestinal mucosal epithelial cells were necrotic and sloughed into the lumen in the *C. perfringens* + PBS challenge group. Some intestinal glandular epithelial cells became necrotic and detached, but others disappeared completely. The structures of other layers remained intact, and necrotizing enteritis was observed (Figure 11s). Pathological changes in the ileum were consistent with those observed in the jejunum (Figure 11v). No obvious pathological lesions were observed in the *C. perfringens* + CPP lysate group or the blank control group (Figure 11t,u,w,x).

The pathological changes observed in the *C. perfringens* + PBS challenge group were interpreted as direct damage to multiple organs mediated by toxins and as organ dysfunction induced by inflammatory responses. The organizational structure in the *C. perfringens* + CPP lysate group was identical to that in the blank control group, and no obvious pathological damage was observed. These findings provide histopathological evidence supporting the prophylactic potential of phage vB_CppII_Hohhot against *C. perfringens* infection (Figure 11).

## 4. Discussion

*C. perfringens* are highly pathogenic, anaerobic, spore-forming bacterium that presents a major threat to animal husbandry. It causes diseases such as necrotic enteritis and enterotoxemia, which often result in high mortality in calves [36]. The improper long-term use of antibiotics not only drives the spread of drug-resistant strains but also disrupts intestinal microbial homeostasis, indicating an urgent demand for safe and effective new prevention and control strategies [16,18]. In this study, a newly isolated *C. perfringens* phage, vB_CppII_Hohhot, was isolated and characterized from wastewater samples collected at a large-scale ranch in Inner Mongolia. Systematic characterization of its physicochemical properties, genomic features, and prophylactic efficacy in a mouse model provides a proof-of-concept for this newly isolated phage as a potential biocontrol agent against *C. perfringens*.

The isolated phage vB_CppII_Hohhot was assigned to the class *Caudoviricetes*. An icosahedral head and a helical tail were observed as its typical structural features. These traits allowed the phage to penetrate the *C. perfringens* cell wall through tail contraction, and lytic efficiency was enhanced by this mechanism [16,37]. Physicochemical analysis was conducted. The phage was found to remain stable across pH 3 to 11 (the test scope examined the tolerance of the bacteriophage under extreme acidic or alkaline conditions, covering the pH range within the stomach), and peak infectivity was observed at 37 °C to 40 °C. with the phage titer falling to 0 PFU/mL at temperatures above 45 °C. These properties were consistent with the natural infection environment of *C. perfringens* The phage tolerated acidic conditions in the animal gastrointestinal tract, such as gastric pH 3 to 4, and proliferated efficiently at physiological body temperature. The host-range assessment was limited to a single C. perfringens type A strain and a small panel of non-target bacteria; therefore, broader activity against diverse isolates requires further investigation.

An optimal MOI of 0.1 was determined. Efficient infection was achieved at low phage doses, and the costs for practical application were reduced. An explosive proliferation phase was observed from 40 min, and a significant reduction in bacterial density was achieved within 14 h. Under the conditions of the dissociation curve measurement, a complete elimination effect was not achieved by the isolated lytic phages. While the phage exhibited potent lytic activity in vitro (Figure 2C), the lysis curve assay (Figure 6B) showed a plateau, with bacterial density remaining above zero at 24 h. This discrepancy likely stems from multiple factors: intermittent oxygen exposure during sampling may have suppressed the obligate anaerobe C. perfringens; oxygen levels are known to modulate phage latent period, burst size, and resistance development; and the emergence of resistant variants cannot be ruled out. Nevertheless, the early-phase (0–6 h) reduction in bacterial burden was sufficient to attenuate tissue damage in the prophylactic mouse model, indicating that functional pathogen clearance—rather than absolute sterilization—is the relevant efficacy indicator in this setting. And in the future lysis curve assays should incorporate parallel phage titer monitoring to better interpret the observed OD_600nm_ profiles.

Whole-genome sequencing was performed. The genome of phage vB_CppII_Hohhot was determined to be 53,225 bp in length, and the G + C content was 34.14%. Screening against the CARD and VFDB revealed no known antibiotic resistance or virulence genes based on current database searches and thresholds, alleviating major safety concerns that have been raised for certain phages carrying risk-associated genes. However, this safety assessment is limited by the presence of 39 uncharacterized ORFs with no known homologs, whose potential functions remain undetermined. A core functional gene was identified, encoding N-acetyl-cellulocysteinyl-L-alaninamidase (lysozyme), which mediated lysis of the host bacteria. This enzyme directly degraded peptidoglycan in the bacterial cell wall, and its bactericidal activity was retained even after the phage lytic cycle had ended. This mechanism was consistent with previous reports on the cpp-lys lysin [15] and LysCPAS15 [38]. COG and GO annotations showed that a high proportion of the phage genes were associated with DNA replication and nucleotide metabolism, which indicated robust mechanisms for genetic material maintenance and evolution and provided a molecular foundation for efficient proliferation. Thirty-nine genes had no matches to known sequences, and these genes may encode components related to the lytic mechanism or host adaptation.

The pH tolerance range of phage vB_CppII_Hohhot was compared with those reported in existing studies. Zhao et al. [15] reported that the cpp-lys endolysin remained stable at pH 4 to 9, and Wu et al. [17] observed stability at pH 5 to 9 for phage vB_CpeP_15N3. In contrast, vB_CppII_Hohhot retained activity at pH 3. Thermal stability was also examined, and a peak activity of the phage was observed at 37 °C to 40 °C, which corresponded to the body temperatures of target animals such as cattle and mice. Some phages, including cpp-lys, were reported to retain activity above 50 °C [15,38], and stability under physiological body temperature conditions more relevant for agricultural applications.

In the physicochemical property assays, temperature and pH gradient designs were used. Extreme real-world application scenarios, such as high temperatures in summer pastures or intestinal pH fluctuations in stressed animals, were not fully simulated with these designs. Growth curves and lysis curves were determined only under optimal MOI and temperature conditions. The effects of fluctuating environmental factors on phage activity were not investigated. Therefore, key variables encountered in practical agricultural applications were not examined, including the use of phages in poultry and swine farming to control necrotic enteritis, as a feed additive to reduce C. perfringens colonization, or for environmental disinfection in livestock facilities.

FastANI analysis was performed on vB_CppII_Hohhot. The average nucleotide identity (ANI) between this isolate and 11 known Clostridium phages was found to range from 97.2% to 98.2%, and nearly 100% alignment coverage was observed. The unique genetic makeup of this phage may endow it with specific host adaptability. Functional characterization was performed in the present study. The prophylactic potential of this phage was validated in a murine challenge model. The therapeutic efficacy was not assessed, and this aspect was indicated for future investigation. This dual consideration, covering both preventive and possible therapeutic applications, had rarely been addressed by a single phage in previous reports. For instance, LysCPAS15 was primarily investigated for foodborne antibacterial activity [38], but ΦCPV1 was mainly studied for therapeutic applications [37], and few studies had combined both functionalities. Genomic analysis revealed no known antibiotic resistance or virulence genes based on current database searches and the thresholds described in the Methods, alleviating major safety concerns that have been raised for certain phages carrying risk-associated genes [39], were not applicable to this phage. However, this safety assessment was limited by the presence of 39 uncharacterized ORFs with no known homologs. The potential functions of these ORFs, including possible roles in host recognition, replication regulation, or lytic efficiency, remained undetermined. Optimized oral protective formulations, such as microencapsulation or enteric coating, were not employed in the current study. In the absence of such formulations, gastric acid-mediated phage inactivation could compromise oral delivery efficacy [15,16]. This aspect was indicated for future translational development.

In the mouse infection model, no pathological damage was observed in the *C. perfringens* + CPP lysed group or the blank control group. Alpha-toxin levels in the *C. perfringens* + CPP lysed group showed no significant difference from the blank control group, whereas a highly significant difference was observed compared with the *C. perfringens* + PBS challenge group. These results are consistent with the prophylactic efficacy of phage vB_CpeP_15N3 reported by Wu et al. [17]. The basic biological properties and in vitro lytic activity of phage vB_CppII_Hohhot against *C. perfringens* were preliminarily characterized. Evidence supporting the protective efficacy of this phage in mice was provided by these findings. No overt adverse effects were observed within the experimental framework. The phage was considered a potential antibiotic alternative for livestock, pending further development. Several limitations of the current study were identified. The intraperitoneal injection route enabled controlled evaluation of in vivo bacterial inactivation, but it did not replicate the intestinal environment relevant to *C. perfringens*-associated enteric diseases. Critical processes such as gastrointestinal colonization, luminal survival, microbiota interactions, and oral delivery were therefore bypassed. In addition, the effects of the phage on the gut microbiota were not examined. Phage administration can modulate the gut microbial community in a manner that varies with phage species, dosing regimen, and host factors. Further research, including metagenomic profiling in animal models or clinical specimens, was indicated to confirm the in vivo safety of this phage and its influence on intestinal microecology. The present findings were interpreted as a proof-of-concept demonstration that the phage retained lytic activity in vivo under controlled conditions, but not as direct validation of efficacy against enteric disease.

The initial phage titers used for in vitro and in vivo experiments were described (Section 2.2.12), and phage titers were monitored at multiple time points during the in vitro one-step growth curve assay (Figure 5). However, dynamic monitoring of phage titers during the in vivo experiment was not performed. Phage stability, tissue distribution, and clearance in the mouse model remained uncharacterized. A full assessment of phage pharmacokinetics and its correlation with the observed protective efficacy was therefore limited. Future studies incorporating longitudinal phage titer monitoring in blood and target tissues were indicated to better interpret the in vivo protective effects and to guide dose optimization. Future studies employing oral infection and oral phage delivery systems, such as intestinal loop assays or gavage-based challenge models, were indicated to evaluate the application potential of this phage in livestock settings.

## 5. Conclusions

A newly isolated phage against C. perfringens, designated vB_CppII_Hohhot, was isolated from wastewater collected from dairy farms in Inner Mongolia and characterized for its biocontrol potential. The phage was phylogenetically assigned to the class *Caudoviricetes* and was observed to possess a morphology typical of tailed phages, with an icosahedral head and a helical tail. Robust stability was demonstrated across pH 4–11 and temperatures of 25–40 °C. At an optimal MOI of 0.1, the phage caused a significant reduction in host bacterial density within 14 h, demonstrating its lytic activity against *C. perfringens*. Genomic sequencing revealed no known antibiotic resistance or virulence genes based on current database analyses. In vivo experiments showed that the phage effectively alleviated C. perfringens-induced pathological damage in mice, and no adverse effects were detected. On the basis of these findings, vB_CppII_Hohhot is considered a promising candidate for phage-based intervention in livestock; nevertheless, its effects on gut microbiota and long-term safety require further investigation.

## 6. Patents

A novel Clostridium perfringens phage and application thereof. Chinese Patent CN202511728679.0, filed 2025 (pending).

## Data Availability

The original contributions presented in this study are included in the article. Further inquiries can be directed to the corresponding authors.
